# Assessments of iodoindoles and abamectin as inducers of methuosis in pinewood nematode, *Bursaphelenchus xylophilus*

**DOI:** 10.1038/s41598-017-07074-2

**Published:** 2017-07-28

**Authors:** Satish Kumar Rajasekharan, Jin-Hyung Lee, Vinothkannan Ravichandran, Jintae Lee

**Affiliations:** 1 School of Chemical Engineering, Yeungnam University, Gyeongsan, 38541 Republic of Korea; 2 Shandong University–Helmholtz Institute of Biotechnology, School of Life Science, Shandong University, Jinan, P.R. China

## Abstract

*Bursaphelenchus xylophilus* is a quarantined migratory endoparasite known to cause severe economic losses in pine forest ecosystems. The study presents the nematicidal effects of halogenated indoles on *B*. *xylophilus* and their action mechanisms. 5-Iodoindole and abamectin (positive control) at low concentration (10 µg/mL) presented similar and high nematicidal activities against *B*. *xylophilus*. 5-Iodoindole diminished fecundity, reproductive activities, embryonic and juvenile lethality and locomotor behaviors. Molecular interactions of ligands with invertebrate-specific glutamate gated chloride channel receptor reinforced the notion that 5-iodoindole, like abamectin, rigidly binds to the active sites of the receptor. 5-Iodoindole also induced diverse phenotypic deformities in nematodes including abnormal organ disruption/shrinkage and increased vacuolization. These findings suggest the prospective role of vacuoles in nematode death by methuosis. Importantly, 5-iodoindole was nontoxic to two plants, *Brassica oleracea* and *Raphanus raphanistrum*. Henceforth, the study warrants the application of iodoindoles in ecological environments to control the devastating pine destruction by *B*. *xylophilus*.

## Introduction

*Bursaphelenchus xylophilus*, clustered under pinewood nematodes (PWN), is a migratory endoparasitic nematode known to cause severe environmental damage of pine forest ecosystems^1^. The PWNs causes the pine wilt disease (PWD), a disease which is becoming an issue of serious concern in several continents including Asia, and Europe, while in North America, the PWN is known for killing exotic pine species^1, 2^. Pine destruction is a major economic concern and the prospect of global spread is alarming^3^. The majority of pine species affected by PWD are *Pinus densiflora, Pinus sylvestris, Pinus thunbergii*, *Pinus koraiensis, Pinus pinaster, Pinus nigra*, and *Pinus radiate* ^4^. PWD is a dramatic disease that kills pine trees within weeks or few months after infection. Furthermore, the PWD epidemic has prevailed in diverse ecosystems, and thus, resilient infection chains have been established^1^.

*B*. *xylophilus* is a quarantined plant parasitic nematode of the Aphelenchoidoidea superfamily and belongs to clade 10 tylenchid^2, 5^. This nematode is a fungal feeder, reproduces within pinewood tissues, and develops through four different juvenile stages namely, L1, L2, L3, L4 and adults^1, 6^. Under food deprived conditions, *B*. *xylophilus* enters a specialized dauer juvenile stage, boards its vector *Monochamus alternatus* (the sawyer beetle), and is transported to healthy pine trees. In healthy hosts, the nematodes migrate rapidly through plant tissues and feed on parenchymal cells, which leads to a cascade of hypersensitive reactions, pine wilting, and death within a year of infection^1, 7, 8^.

The biological control of *B*. *xylophilus* remained challenging for a long time, and quarantine efforts have been established during the 20th century. The current strategies used to combat PWNs mainly by chemical treatments, which include fumigation of wood materials and nematicide implantation in trunks. The most frequently used nematicides are abamectin and emamectin benzoate, which belong to the avermectin family. These expensive chemicals are highly effective against several nematodes and are also considered environmentally safe^9^. However, it is speculative that the recurrent use of these nematicides will induce selection pressure and almost certainly produce resistant PWNs, as has been demonstrated for several insect pests, such as, *Leptinotarsa decemlineata, Plutella xylostella* and the nematodes *Trichostrongylus colubriformis* and *Ostertagia circumcincta* progressively developed resistance to abamectin^10–12^. Hence, patterns of resistance should be scrutinized regularly and screening of nematicides should be continuously performed to identify alternative cost-effective and eco-friendly measures to combat PWD. During recent decades, several authors have suggested the use of plant extracts, essential oils, and volatiles, as nematode control agents^13–16^.

Recently, we demonstrated the nematicidal effect of indole (an intercellular and interkingdom signaling molecule) on *Caenorhabditis elegans* ^17^. Indole is a widespread intracellular signal in microbial ecology and governs multiple functions that influence microbial physiology, spore formation, plasmid stability, drug resistance, biofilm formation, and virulence^18, 19^. The effects of indole and its derivatives have not been examined in other pathogenic nematodes. In the present study, we studied the nematicidal effects of 34 indoles on *B*. *xylophilus*, and investigated the action mechanism of the most potent, 5-iodoindole, using microscopic techniques, time-lapse imaging, molecular docking experiments, and assessed its toxic effects on plants using a seed germination test.

## Results

### Indole and halogenated indoles restrain *B*. *xylophilus* survival

Previously, indole at high concentrations (>1.0 mM) was reported to have nematicidal on *C*. *elegans* ^17^. Following treatment of *B*. *xylophilus* (mixed developmental stages) with indole or 33 different indole derivatives at 1 mM, the mortality rates of *B*. *xylophilus* were measured by counting live and dead nematodes in control and treated groups. Five indoles exhibited notable nematicidal activity; non-treated controls showed 95 ± 7% survival after 24 h. Of the 34 indoles tested, 5-iodoindole and 4-fluroindole at 1 mM produced 100% mortality, while 5, 6-difluoroisatin, methyl indole-7-carboxylate, and 7-iodoindole at this concentration had mortality rates of approximately 50% (Table 1).

**Table 1:** Effect of indole and indole derivatives (1 mM) on the survival of *B*. *xylophilus*.

Compounds (Purity, %)	Survival (%)	Compounds (Purity, %)	Survival (%)
None	95 ± 7	5-Fluoroxiindole (98%)	78 ± 5
Indole (≥99%)	80 ± 7	7-Fluoroindole (97%)	86 ± 19
Indole-3-acetamide (98%)	96 ± 5	5-Fluoroindolin-2,3-dione (98%)	96 ± 6
Indole-3-acetonitrile (98%)	92 ± 11	7-Fluoroindoline-2,3-dione (97%)	95 ± 7
Indole-3-acetic acid (98%)	95 ± 7	8-Fluoroquinoline (98%)	100
Indole-3-butyric acid (≥99%)	88 ± 16	7-Formylindole (97%)	89 ± 15
Indole-3-carbinol (98%)	85 ± 10	7-Hydroxyindole (97%)	89 ± 6
Indole-3-propionic acid (≥97%)	92 ± 1	**5-Iodoindole (98%)**	**0**
7-Azaindole (98%)	75 ± 9	**7-Iodoindole (97%)**	**46 ± 5**
1-BOC-7-methylindole (95%)	78 ± 4	3,3′-Methylene bisindole (98%)	86 ± 6
5-Bromo-3-iodo-7-azaindole (97%)	95 ± 6	**Methyl indole-7-carboxylate (98%)**	**43** **±** **4**
7-Bromoindole (96%)	90 ± 6	7-Methylindole (97%)	86 ± 1
7-Chloroindole (99%)	82 ± 12	7-Methoxyindole (97%)	87 ± 4
5,6-Difluoroisatin (96%)	51 ± 13	7-Nitroindole (97%)	71 ± 3
**4-Fluoroindole (97%)**	**0**	7-(Trifluoromethyl) isatin (98%)	90 ± 14
5-Fluoroindole (98%)	83 ± 9	Melatonin (≥99.5%)	89 ± 5
6-Fluoroindole (98%)	87 ± 5	Serotonin (95%)	89 ± 7

Important changes are shown in boldface.

**Figure 1:**
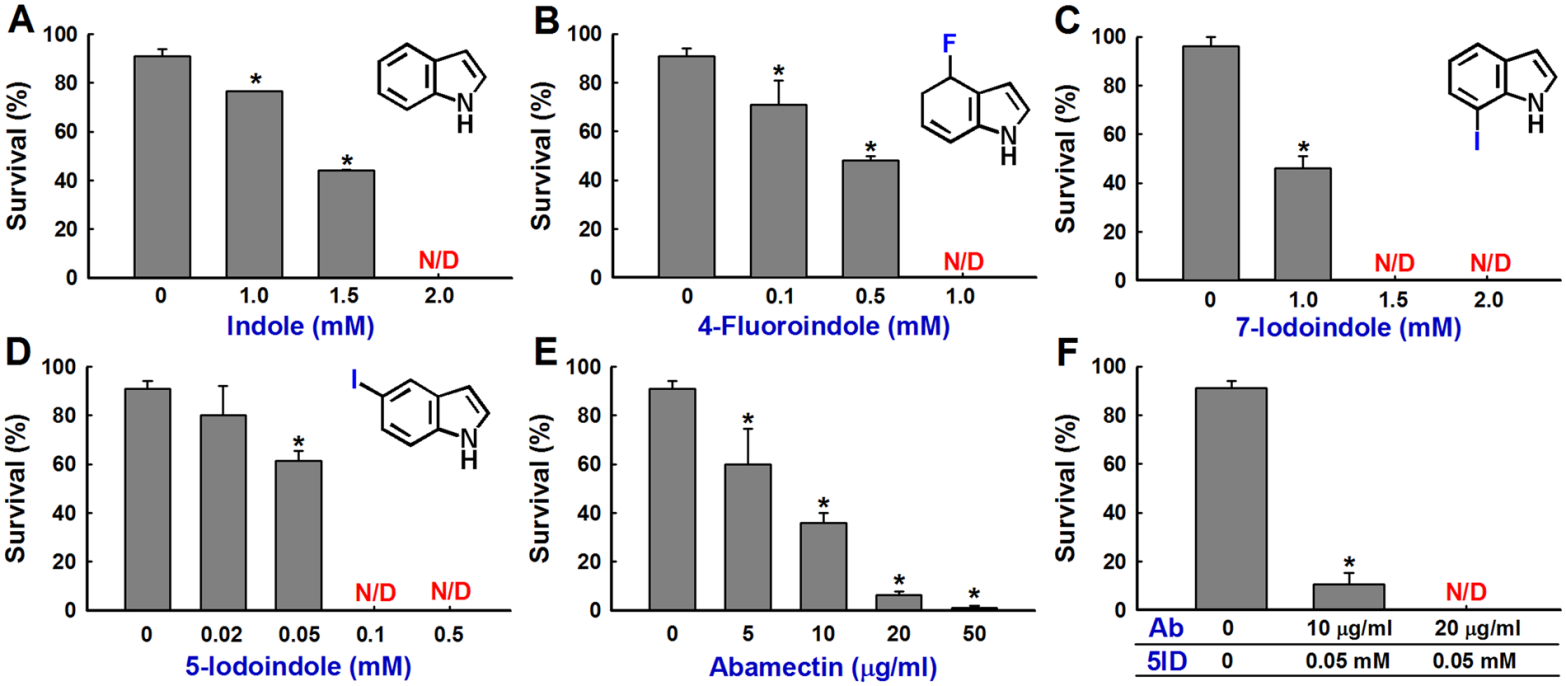
Nematicidal effects of indoles and abamectin on *B*. *xylophilus* survival. Dose dependent effects of (**A**) indole, (**B**) 4-fluoroindole, (**C**) 7-iodoindole, (**D**) 5-iodoindole, and (**E**) abamectin on the survival of *B*. *xylophilus*. (**F**) Effect of 5-iodoindole in combination with abamectin on *B*. *xylophilus* survival. Ab indicates abamectin and 5ID indicates 5-iodoindole. The graph shows the means ± SEMs of three repetitions. *P < 0.05 vs. the non-treated control; and N/D indicates not detected.

Indole exhibited significant nematicidal activity at 1.5 mM and complete killing at 2 mM (Fig. 1A). Further assays were performed to check nematode survival rates at lower concentrations (0, 0.02, 0.05, 0.1 and 0.5 mM). 4-Fluroindole showed nematode killing (approximately 50%) at 0.5 mM (Fig. 1B) while 7-iodoindole achieved complete killing at 1.5 mM (Fig. 1C). Interestingly, 5-iodoindole showed most significant decline in the survival of *B*. *xylophilus* at 0.05 mM and killed all the nematodes at 0.1 and 0.5 mM (Fig. 1D). Treatments with 5-iodoindole (0.05 mM) and abamectin (10 µg/mL) resulted in 61 ± 2% and 36 ± 4% survival of *B*. *xylophilus*, respectively (Fig. 1D and E). Hence, 5-iodoindole and the commercial nematicide, abamectin, were used for conducting additional experiments with *B*. *xylophilus*.

5-Iodoindole at 0.05 mM (12.15 µg/mL) and abamectin (5 µg/mL) had similar effects on nematode survival (Fig. 1D and E). As was expected, abamectin dose-dependently killed *B*. *xylophilus* with maximum inhibition at 50 µg/mL, although significant declines in nematode survival were noted at 10 and 20 µg/mL (Fig. 1E), which concurs with previous results^20^. Enhanced activity was observed when 5-iodoindole and abamectin were used in combination at 0.05 mM and 10 µg/mL respectively (~11 ± 5% survival) (Fig. 1F). Complete killing was achieved when 5-iodoindole (0.05 mM) and abamectin (20 µg/mL) was used in combination. Previously, it was shown that abamectin exhibited high nematicidal against *B*. *xylophilus* at 12.53 µg/mL which was considered as the LD_90_ value^3^, hence further experiments were conducted with 10 µg/mL of abamectin as similar effect was observed at this concentration.

### 5-Iodoindole and abamectin both interrupted thrashing movements and disrupted reproductive traits in *B*. *xylophilus*

**Figure 2:**
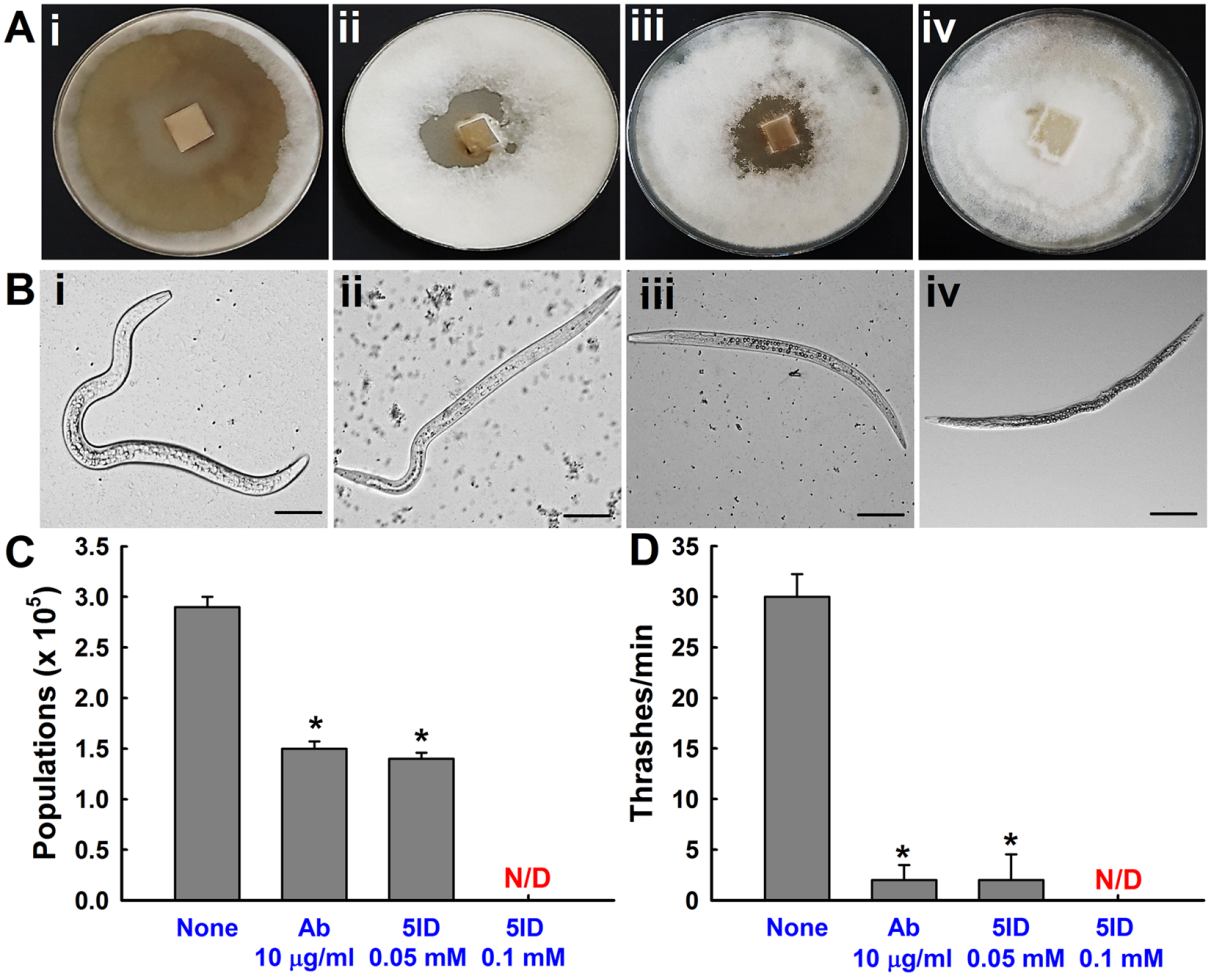
Influence of 5-iodoindole on the reproductive and locomotor activities of *B*. *xylophilus*. (**A**) Areas of *B*. *cinerea* mold eaten by *B*. *xylophilus* after 8 d, showing differences in inhibitory activities [(i) none, (ii) abamectin (10 µg/mL), (iii) 5-iodoindole (0.05 mM), and (iv) 5-iodoindole (0.1 mM)]. (**B** and **D**) Effects of 5-iodoindole and abamectin on the thrashing rate of *B*. *xylophilus* [(i) none, (ii) abamectin (10 µg/mL), (iii) 5-iodoindole (0.05 mM) and (iv) 5-iodoindole (0.1 mM)]. (**C**) Effects of 5-iodoindole and abamectin on nematode population numbers. Ab indicates abamectin and 5ID indicates 5-iodoindole. The graph shows the means ± SEMs of two trials with six repetitions. *P < 0.05 vs. the non-treated control; and N/D indicates not detected.

To characterize the influences of 5-iodoindole and abamectin on fecundity and reproductive activities, we counted the nematode populations. *B*. *xylophilus* was initially inoculated on the surfaces of fully grown *Botrytis cinerea* mycelia. Mycelia were consumed by nematodes in control plates as evidenced by a clear zone (Fig. 2Ai), while in the plates treated with 5-iodoindole (0.05 mM) or abamectin (10 µg/mL) clearance zones were reduced similarly, reflecting similar efficacy of both the chemicals at similar concentrations (Fig. 2Aii and iii). Notably, *B*. *xylophilus* in the presence of 5-iodoindole at 0.1 mM did not consume mycelia at all (Fig. 2Aiv). In addition, nematode populations in plates were counted and 5-iodoindole (0.05 mM) or abamectin (10 µg/mL) were both found to reduce nematode numbers to 1.4 × 10^5^ ± 0.1 and 1.5 × 10^5^ ± 0.1 respectively, compared with non-treated controls (2.9 × 10^5^ ± 0.1) (Fig. 2C).

Nematodes are characterized by their rapid special movements and violent thrashing behaviors which are under the control of somatic motor neurons^21^. Nematode thrashing frequencies were significantly reduced by 5-iodoindole (0.05 mM) (2 ± 1.50 thrashes/min) and by abamectin (10 µg/mL) (2 ± 1.25 thrashes/min) (*P* < 0.01) as compared with non-treated controls (~30 ± 2.25 thrashes/min) (Fig. 2B and D). At 0.1 mM, 5-iodoindole completely abolished thrashing (Fig. 2D). Time-lapse imaging showed that the movements of surviving *B*. *xylophilus* exhibited reduced locomotive behavior in 5-iodoindole and abamectin treated nematodes (Fig. 2B and Supplementary Fig. S1).

### 5-Iodoindole and abamectin suppressed embryonic lethality, fecundity, and *B*. *xylophilus* L2s development

**Figure 3:**
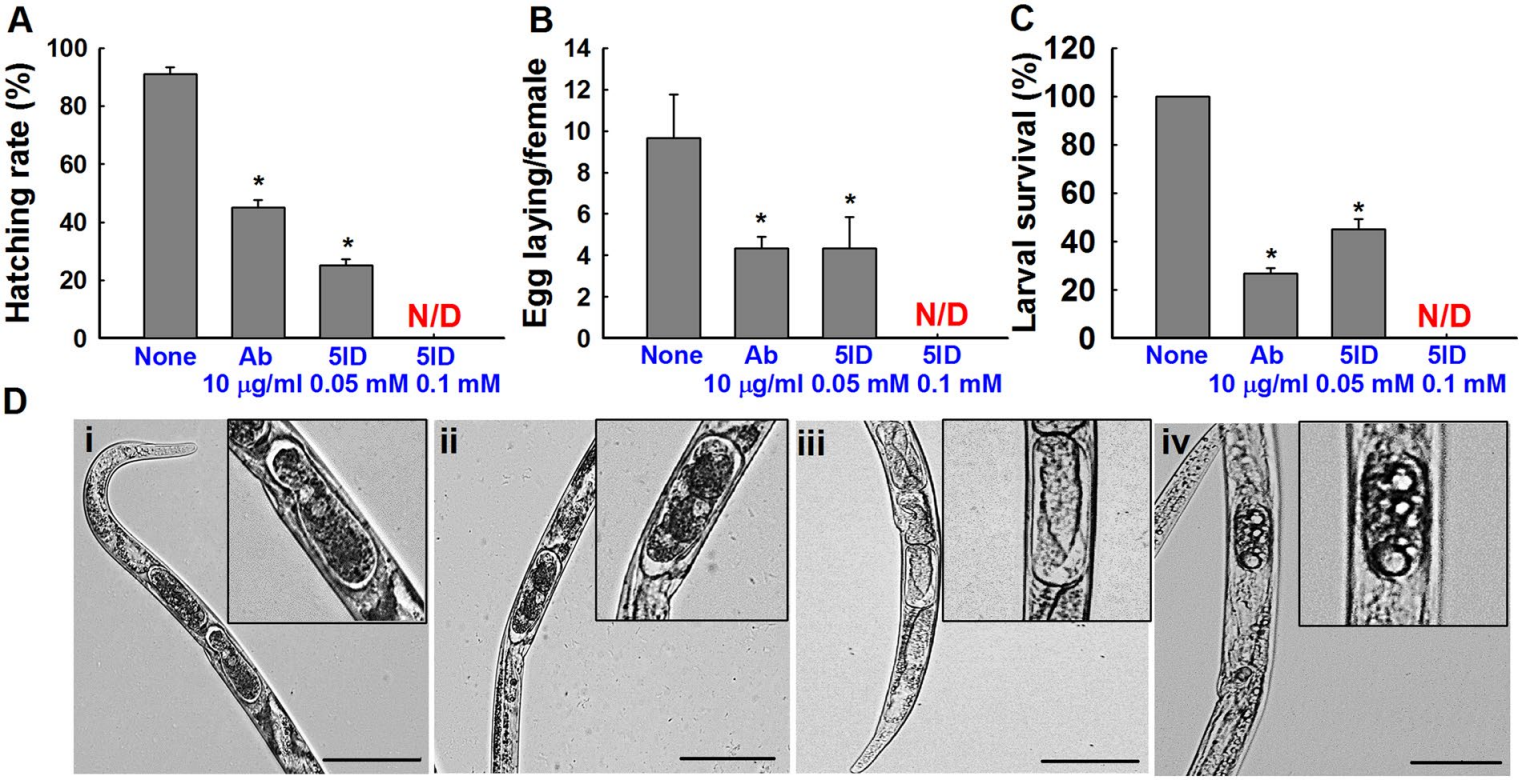
Effects of 5-iodoindole on egg hatching, egg deposition and juvenile mortality. (**A**) Effects of 5-iodoindole and abamectin on cumulative hatching rates after 24 h, (**B**) eggs laid per female nematode after 12 h, (**C**) L2 survival after treatment for 24 h, and (**D**) representative images of female nematodes showing eggs [(i) none, (ii) abamectin (10 µg/mL), (iii) 5-iodoindole (0.05 mM) and (iv) 5-iodoindole (0.1 mM)]. The graphs (A and C) shows the means ± SEMs of two trials with six repetitions whereas graph B shows the means ± SEMs of three repetitions. *P < 0.05 vs. the non-treated controls; N/D indicates not detected; Scale bars = 50 µm.

Embryonic lethality, juvenile lethality and lack of fecundity markedly affected the final populations of parasitic nematodes. 5-Iodoindole (0.05 mM) or abamectin (10 µg/mL) markedly inhibited *B*. *xylophilus* egg hatching (Fig. 3A). Non-treated controls showed a hatch rate of 91 ± 2%, whereas 5-iodoindole at 0.05 mM reduced hatch rate to 25 ± 2%, abamectin at 10 µg/mL also significantly reduced the hatch rate to 44 ± 3% (*P* < 0.05) (Fig. 3A). 5-Iodoindole (0.1 mM) treated L2 eggs failed to hatch (Fig. 3A). Furthermore, 5-iodoindole and abamectin adversely effected the fecundity of adult female nematodes (Fig. 3B and D); control females laid 10 ± 2 eggs in 12 h but treatment with 5-iodoindole (0.05 mM) or abamectin (10 µg/mL) reduced this to 4.3 ± 0.5 and 4.3 ± 1.5 eggs, respectively (Fig. 3B).

Exposure of *B*. *xylophilus* L2s to 5-iodoindole (0.05 mM) drastically reduced *B*. *xylophilus* survival and 5-iodoindole (0.1 mM) eradicated the entire L2s (Fig. 3C). Microscopic visualization also revealed the inability of the gravid adult to lay eggs, leading to accumulation of eggs within the worms (Fig. 3D). L2 stage eggs were also observed inside the worms suggesting internal molting (Fig. 3Diii).

### 5-Iodoindole elicited morphological defects in *B*. *xylophilus* and caused methuosis

**Figure 4:**
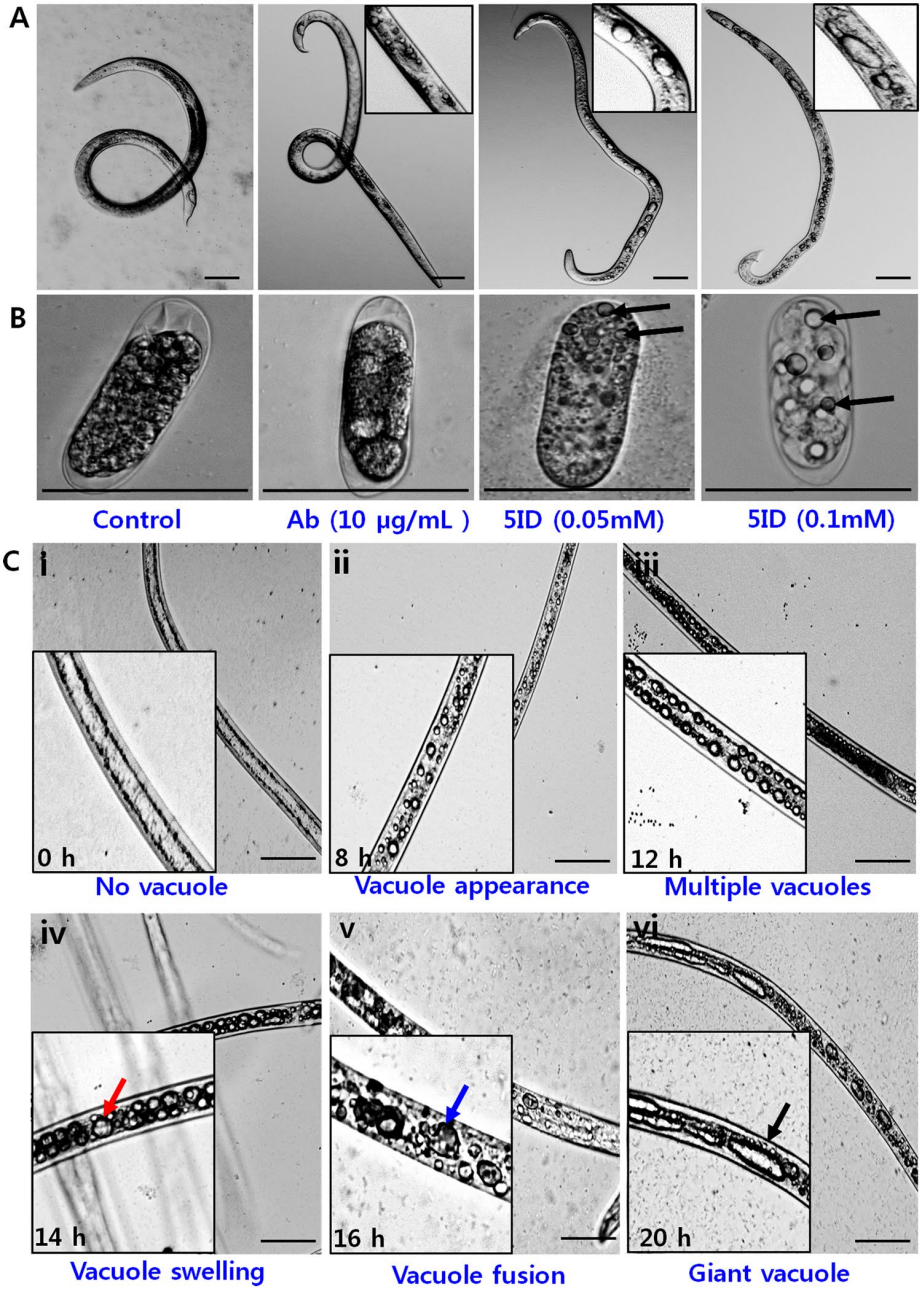
Effect of 5-iodoindole on vacuolization and methuosis in *B*. *xylophilus*. (**A**) Effects of abamectin and 5-iodoindole on adult male nematodes, (**B**) L1 stage nematode egg, and (**C**) Methuosis in *B*. *xylophilus*, (i) no vacuoles were observed at 0 h, treatment resulted in (ii) vacuolar appearance, (iii) multiple vacuole accumulation, (iv) vacuolar swelling, (v) vacuole fusion, and (vi) the formation of giant vacuoles. Red arrow indicates vacuole swelling, blue arrow indicates vacuolar fusion, and black arrow indicates a giant vacuole. Scale bars = 50 µm.

The accumulation of fluid due to osmotic imbalance results in cellular swelling followed by vacuolar swelling in other animals^22–24^. This hydropic change is a result of reversible changes in response to cell injury^25, 26^. Several morphological changes were visualized in 5-iodoindole treated nematodes and their eggs, but not in abamectin or in non-treated controls. Treatment with 5-iodoindole (0.05 or 0.1 mM) resulted in rapid vacuolations in nematode bodies, which resulted in the production of several medium-to-large sized vacuoles (Fig. 4A). Interestingly, multiple giant vacuoles were observed also in nematode eggs (Fig. 4B) and L1 eggs (Supplementary Fig. S2). Three different developmental stages (L2 juveniles, a mixture of L3/L4, and adult nematodes) were treated separately with 5-iodoindole (0.1 mM), which also revealed multiple vacuoles (Supplementary Fig. S3). Furthermore, eggshell membranes, in which oocyte-to-embryo transition takes place, were significantly disrupted by 5-iodoindole (0.1 mM) (Supplementary Fig. S2). The presence of multiple vacuoles in embryos in L1 and L2 eggs showed that 5-iodoindole disrupted the osmotic barrier properties of eggshells.

Giant vacuoles were also observed in dead nematodes treated with 5-iodoindole (0.1 mM), and were mostly localized at terminal ends (Supplementary Fig. S4iv and vi). Along with giant vacuoles, 5-iodoindole also caused obvious internal organ damage, which was associated with the presence of several void spaces in head region, body parts, and tail region (Supplementary Fig. S4iv,v and vi). Large vacuoles and voids were not observed in non-treated controls or abamectin treated worms (Supplementary Fig. S4i,ii and iii).

In addition, the study describes the sequential process of methuotic death in *B*. *xylophilus* (Fig. 4C). Methuosis is a non-apoptotic type of cell death associated with the accumulation of prominent cytoplasmic vacuoles^27^. Morphological defects observed in *B*. *xylophilus* appeared to be well correlated with the mechanism of methuosis. Microscopic imaging at different times revealed that giant vacuoles formed after 20 h of 5-iodoindole (0.1 mM). Minute vacuoles were observed after treatment for 8 h and numbers increased at 12 h. At 14 h, several large vacuoles were observed. After 12 to 16 h of treatment, several fused vacuoles were distinctly visible, suggesting that vacuolar fusion underlay the mechanism of methuosis. At 20 h, several giant vacuoles were observed throughout nematodes. These observations constitute the first report of methuosis in nematodes (Fig. 4C).

**Figure 5:**
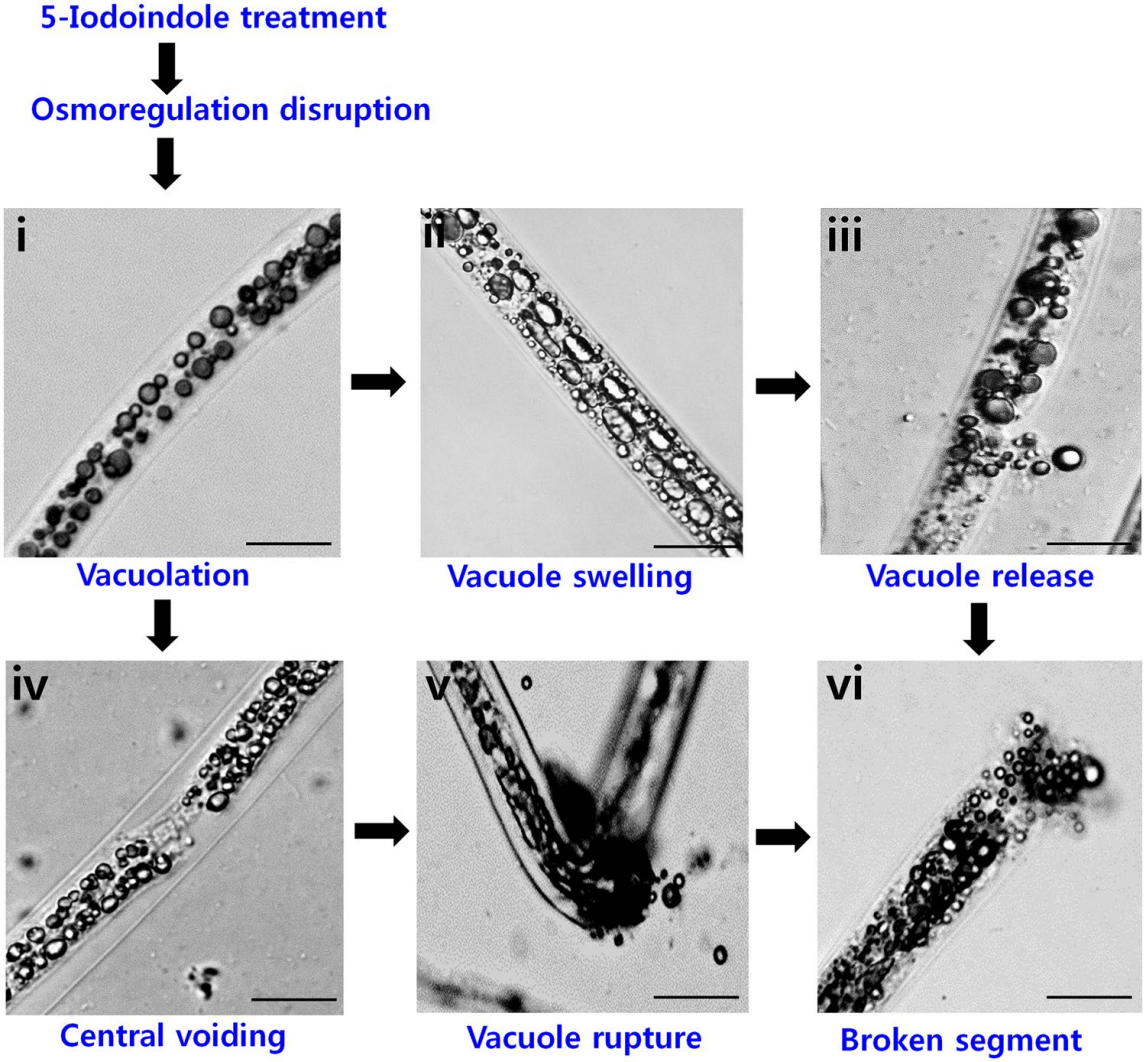
Proposed mechanism of vacuole formation and the sequence of events leading to nematode death. 5-Iodoindole treatment resulted in fluid accumulation and the disruption of osmoregulation, a process which triggers RCI. RCI resulted in (**i**) rapid vacuolation, (**ii**) vacuolar swelling, and (**iii**) vacuole release. In one model, we propose and show that (**iv**) vacuoles aggregated or align in opposite directions creating a large central void, which further (**v**) ruptured, and (**vi**) released the vacuoles to the external environment. Scale bars = 50 µm.

Vacuolar aggregation and rupture was also noted in 5-iodoindole treated nematodes (Fig. 5), which was visualized by nematode bending and vacuole release into the external environment. Vacuole rupture was also noted in egg shell membranes, which are usually left intact by the L2s during hatching (Supplementary Fig. S2). These observations support the involvement of fluid accumulation and disruption of osmoregulation, and reversible cell injury (RCI) in the processes of vacuolization and methuosis (Fig. 5).

### Indole-iodine complex was crucial for vacuolization and methuosis

Anticipating that iodine played a role in the observed vacuole formation, we examined the nematode killing activities of sodium iodide (NaI) and potassium iodide (KI). However, neither affected nematode survival nor vacuole formation at concentrations (0.1, 0.5, or 1 mM) (Supplementary Fig. S5), although KI at 1 mM had a slight nematicidal effect. On the other hand, 7-iodoindole (1 or 2 mM), like 5-iodoindole, produced multiple vacuoles and structural deformities (Supplementary Fig. S6). Both iodoindoles exhibited similar phenotypic traits in *B*. *xylophilus*, whereas NaI and KI did not. Interestingly, indole did not induce vacuole formation in *B*. *xylophilus* at the concentrations tested (data not shown). Hence, the result substantiates that indole-iodine complex is responsible for eliciting vacuolization and methuosis in *B*. *xylophilus*.

### Molecular modelling of the binding of 5-Iodoindole with glutamate-gated chloride channel (GluCL)

Abamectin (Avermectin B2) is commercial nematicide that kills parasitic nematodes by binding to GluCL receptor and opening the ion channel^28–31^, thus causing rapid chloride ion influx into cells, leading to membrane hyperpolarization, and subsequent paralysis. Anticipating that 5-iodoindole might bind to GluCL receptor, molecular docking modelling was performed to investigate the binding interactions of several halogenated indoles (2-iodoindole, 3-iodoindole, 4-iodoindole, 5-iodoindole, 6-iodoindole, 7-iodoindole, 4-fluroindole, and indole). As expected, ivermectin, an avermectin derivative and GluCL receptor were found to bind strongly (binding energy: −10.98 kcal/mol) in our docking simulation (Supplementary Fig. S7). Interactions were found to be stabilized by robust backbone hydrogen bonding with leucine 218 of GluCL receptor and by a relatively weak side chain hydrogen bonding with glutamine 219. Abamectin also displayed good binding energy with −6.52 kcal/mol but did not form any side chain or back bone hydrogen bonding (Supplementary Fig. S7).

**Figure 6:**
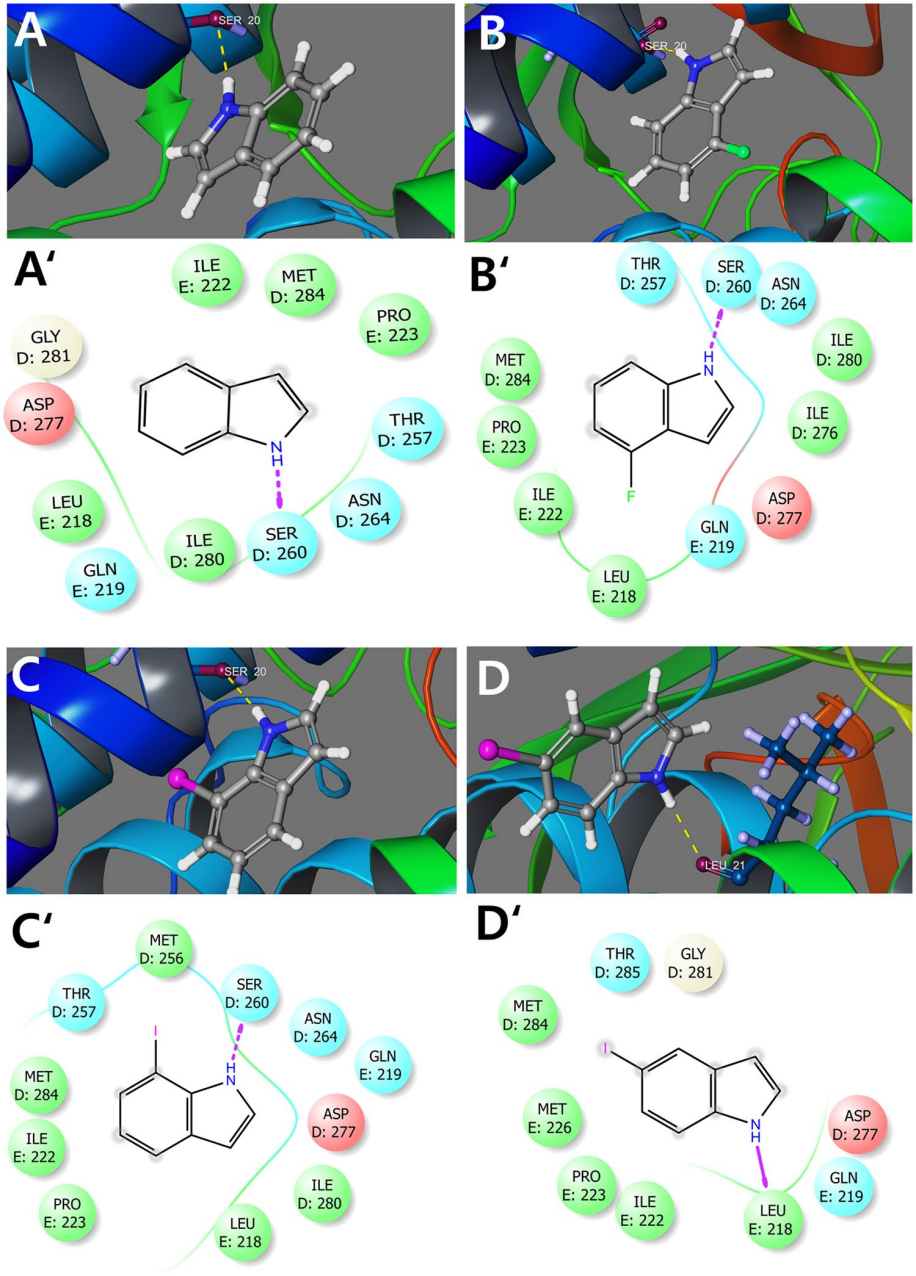
Molecular dockings of indole and halogenated indoles with GluCL. Binding orientations of ligands (**A**) indole, (**B**) 4-fluroindole, (**C**) 7-iodoindole, and (**D**) 5-iodoindole with the active sites of GluCL. The protein is represented by ribbons and backbone hydrogen bonds are shown as dotted yellow lines. (**A′**), (**B′**), (**C′**), and (**D′**) show interactions for respective ligands and surrounding amino acid residues and sidechain hydrogen bonds are shown as dotted pink arrow.

Among the indoles tested for nematicidal activity, 5-iodoindole had the highest glide score of −5.89 kcal/mol followed by 7-iodoindole (−4.48 kcal/mol), 4-fluroindole (−4.33), and indole (−4.03) (Fig. 6). 5-Iodoindole binding was stabilized by strong backbone hydrogen bond with leucine 218 while all the other indole derivatives bonded with side chain hydrogen bonding with serine 260 residue. Of the other iodoindoles simulated, 2-iodoindole had a binding score of −5.248 kcal/mol due to a backbone hydrogen bonding with leucine 218. Other notable bindings were observed by 3-iodoindole (−4.3 kcal/mol), 4-iodoindole (−4.0 kcal/mol) and 6-fluroindole (−2.6 kcal/mol) (Supplementary Fig. S8). Most of the halogenated indoles and indole itself formed bonds with serine 260 except 5-iodoindole and 2-iodoindole. Hydrogen bonding with leucine 218 was found to be an indicator of efficient receptor-ligand binding, as was observed for ivermectin (Supplementary Fig. S7), which supports that 5-iodoindole and 2-iodoindole, like ivermectin binds strongly to the active sites of GluCL receptor via leucine 218 (Fig. 6 and Supplementary Fig. S8). We suggest this binding is crucial for maintaining the open pore structure of GluCL complex, and that by strongly binding with the active sites of GluCL receptor, 5-iodoindole, 2-iodoindole, abamectin and ivermectin thus keep the ion channel open and allow fluid intake.

### 5-Iodoindole has no toxic effect on seed germination

**Figure 7:**
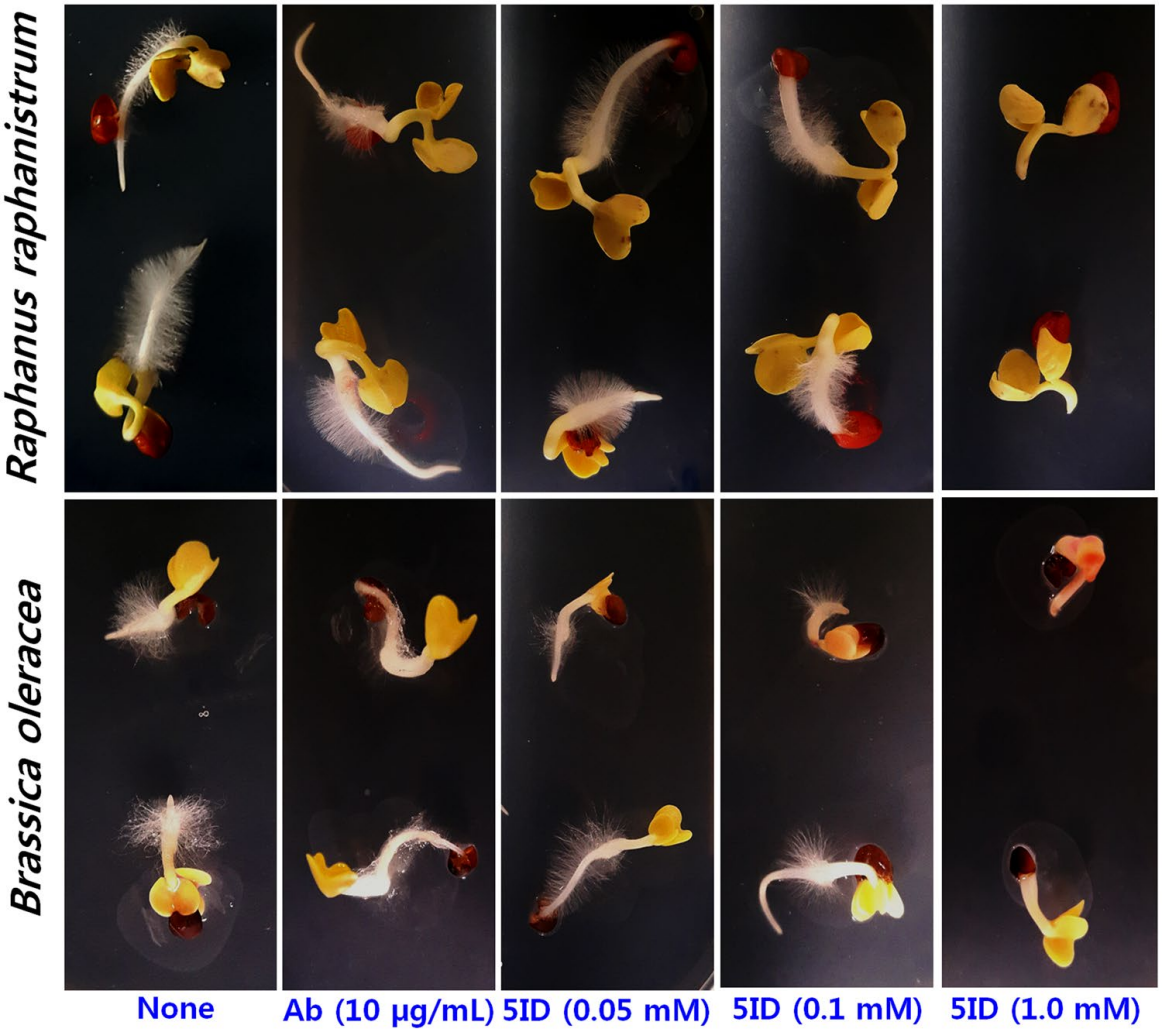
Effect of 5-iodoindole on seed germination. *B*. *oleracea* and *R*. *raphanistrum* seeds on Murashige and Skoog agar medium supplemented with or without abamectin or 5-iodoindole, showing sprouting, germination, and lateral roots. Germination was recorded after incubation for 3 days at 22 °C.

Experiments were conducted to assess the toxic effect of 5-iodoindole on *Brassica oleracea* and *Raphanus raphanistrum* seed germination. 5-Iodoindole (0.05 or 0.1 mM) or abamectin (10 µg/mL) had no or negligible effects on initial sprouting and germination (Fig. 7). Furthermore, no significant difference was observed between the germination rates of non-treated controls or 5-iodoindole or abamectin treated seeds. Effects on primary root elongation and numbers of lateral roots formed were negligible, though lateral root development was slightly delayed by 5-iodoindole at 1 mM (10 times higher than its active concentration). These results suggest that 5-iodoindole, at the tested concentrations, is non-toxic to plant cells and does not hinder plant developmental processes.

## Discussion

This study reports nematode killing by several indoles. Importantly, this is the first report of methuosis (a process induced by accumulation of small vacuoles that progressively fuse into giant vacuoles, ultimately leading to membrane rupture and death) in PWN induced by iodoindoles, which exhibited marked nematicidal properties like the commercial nematicide, abamectin.

Indoles have been previously reported to play multifunctional signaling roles in the contexts of biofilm inhibition/formation, bacterial survival, and pathogenicity in prokaryotes and eukaryotes^19, 32–34^. Recently, the potential therapeutic effects of halogenated indoles, indole alkaloids, and semisynthetic indole derivatives have attracted considerable research interest^35–37^. For example, halogen substituted indoles have been shown to eliminate *Escherichia coli* and *Staphylococcus aureus* persister cells^37^. Further, exploring the potency of halogenated indoles on other species, genus and kingdom is a research importance and the study is a phase to reach the initiative.

In the present study, 5-iodoindole reduced nematode survival even at a concentration as low as 0.05 mM (Fig. 1C). A previous study reported the effect of emamectin benzoate on the fecundity of *B*. *xylophilus* ^38^, and we found similar effects for 5-iodoindole and abamectin (Fig. 1D and E). We show a significant decrease in the population numbers of *B*. *xylophilus* in the population inhibitory tests (Fig. 2A and C), which support the fact that 5-iodoindole effectively controls the reproductive traits of *B*. *xylophilus*. Inhibitory effect of nematicides on thrashing behaviors of PWN have been widely reported^20, 38–40^. Time-lapse imaging revealed 5-iodoindole and abamectin treated nematodes (Fig. 2B,D, and Supplementary Fig. S1) were confined to a specific clock position and were almost immobilized with little or no movement of the body parts.

Numbers of eggs laid per female were diminished by abamectin and by 5-iodoindole (Fig. 3B), and this lead to egg accumulation and internal molting, as evidence by the presence of L2 stage eggs inside females (Fig. 3Diii). The credible reason for decreased egg laying ability of *B*. *xylophilus* might be due to the lower O_2_ concentrations (hypoxia) in the distilled water where the gravid females were tested on, as suggested by Zhenzhen *et al*., 2015^38, 41^. Egg hatchability is one of the most important parameters that affects population numbers and any chemical with high embryonic lethality can be considered a potential nematicide^38^. In the present study, 5-iodoindole and abamectin both effectively reduced the hatchability of *B*. *xylophilus* eggs (Fig. 3A), thus fulfilling the decisive criteria of a prospective nematicides.

Observations of dead nematodes under a microscope after treatment provided insight regarding the mode of action of 5-iodoindole. We identified several minute or small specialized fluid accumulating bodies, vacuoles, in *B*. *xylophilus* (Fig. 4Ai). These tiny vacuoles were frequently found in non-treated groups and abamectin treated group (Fig. 4Aii), which we speculate, is a part of normal biological rhythm in *B*. *xylophilus*. The presence of small to intermediate sized vacuoles in nematodes was reported previously and was also observed in our study as well^42^. However, treatment with 5-iodoindole indicated abnormally large multiple vacuoles in the nematode bodies (Fig. 4Aiii and iv), which we propose as a progressive cause for the mortality.

The presence of vacuoles was previously reported in *Meloidogyne incognita*, the southern root-knot nematode (RKN) known to cause root-knot diseases in plants^43^. The authors also reported severe morphological variations in RKN following treatment with oxalic acid which showed complete destruction of internal organs. Studies have also shown that indole-based chalcone derivatives stimulated a caspase independent form of methuotic cell death in U251 glioblastoma and gastric carcinoma cells associated with striking accumulations of giant cytoplasmic vacuoles^44–47^. We observed similar morphological changes in PWN treated with 5-iodoindole. These changes included the formation of giant vacuoles (Supplementary Fig. S4iv and vi), multiple vacuoles (Supplementary Fig. S4v), internal organ disruption, and organelle shrinkage as evidenced by the presence of large voids within nematodes (Supplementary Fig. S4iv,v, and vi), which were not observed in controls (Supplementary Fig. S4i,ii, and iii). Our findings concur with those of Makoto *et al*., 2000, who observed abnormal vacuole formation and the inhibitions of pumping, movement, and egg laying by *C*. *elegans*, when ectopically expressed with activated *mek-1* (Mitogen-activated protein kinase kinase) cDNA^42^. These findings might explain why in our experiments PWN displayed no thrashing or sinusoidal locomotion (Supplementary Fig. S1).

Here, we propose a mechanism for 5-iodoindole induced nematode death based on reversible cell injury (RCI) and methuosis (Figs 4C and 5). Hydropic changes such as swelling and vacuolar degeneration are indicators of RCI and methuosis and are manifested by giant vacuoles in cytoplasm^48, 49^. RCI impedes energy production either by reducing ATP production, by inducing ATPase pump failure, or by damaging the cell membrane and causing the rapid influx of Na^+^, Ca^2+^ and water^50–52^. Fluid accumulation within cytoplasm due to Ca^2+^ and water influx triggers intracytoplasmic vacuolation in animal cells^53^. Interestingly, this mechanism of cell injury is reversible provided the injury is short-lived and the cell starts producing ATP within a certain time frame, but if the injury endures or the condition worsens, cell death follows^54^. Our observations indicate that 5-iodoindole treated nematodes were unable to return to normal biogenesis after exposure to a stressful environment.

The methuosis phenotype induced by 5-iodoindole in *B*. *xylophilus* might be attributed due to the presence of iodine and its molecular disposition since 7-iodoindole inhibited *B*. *xylophilus* less than 5-iodoindole (Table 1 and Supplementary Fig. S6). These findings are in partial agreement with a study by Maltese *et al*., 2014, who reported shifting the nitrogen of the pyridinyl moiety from a para to meta position in indole eliminated vacuolization, growth inhibition, and cytotoxicity in U251 cells, which shows that molecular interactions with specific active sites in proteins are crucial^27, 44, 45^. The interactions observed between indole or halogenated indoles and GluCL receptor in the present study also supports this perception, as 5- and 2-iodoindole were found to bind more strongly with GluCL receptor than the other indoles examined (Fig. 6 and Supplementary Fig. S8). Iodine positioned at the second or fifth positions of indole were found to bind via backbone hydrogen bonding with leucine 218 of GluCL receptor, whereas the other halogenated indoles and indole itself formed weak side-chain hydrogen bonds with serine 260 (Fig. 6). Hence, we hypothesize positioning of the halogen plays a prominent role in the induction of vacuolar degeneration, and that the strong binding of 5-iodoindole keeps the ion channel open allowing rapid fluid influx and vacuolar outburst. However, detailed mechanism responsible of the effects of 5-iodoindole remains to be determined.

Analysis of the toxic effects of 5-iodoindole on plants is indispensable before its practical use. Our seed germination experiments indicate 5-iodoindole at the tested concentrations does not adversely affect seed germination or subsequent developmental processes (Fig. 7). Henceforth, the study warrant the application of 5-iodoindole in ecological environment to control the devastating pine destruction by *B*. *xylophilus*.

Previous reports have established that indole based therapies provide potential means of addressing antibiotic resistance and cancer progression^55^. Furthermore, indoles serve as promising scaffolds for drug development due to their antibacterial, anticancer, anti-oxidant, anti-inflammatory, anti-diabetic, antiviral, antiproliferative, and antituberculosis activities^56, 57^. The present study is the first to suggest the potential use of iodoindoles as antiparasitics or anthelmintics.

Abamectin was discovered three decades ago, was honored with Nobel Prize in 2015 and its use as anthelmintic is still going strong. However, the rapid evolution of abamectin resistance in nematodes and pests requires an alternative, inexpensive, ecofriendly strategy to combat pine tree infection by PWN. This study also reports a mechanism for *B*. *xylophilus* killing by 5-iodoindole and the negligible toxicity of 5-iodoindole to plant cells, which augurs well for its future commercial applications.

## Methods

### Ethics statement

All the experiments were approved by the Ethical Committee of Yeungnam University, Gyeongsan, Korea and the methods were carried out as per the guidelines of the Ethical Committee of Yeungnam University.

### Pinewood nematode, fungal strain and chemicals

The pinewood nematode (PWN), *B*. *xylophilus*, and the mold, *Botrytis cinerea*, were generous gifts from Professors Hanhong Bae and Kwang-Hyun Baek at Yeungnam University. The nematodes were grown on *B*. *cinerea* as feed^58^. *B*. *cinerea* was cultured and maintained in potato dextrose agar plates for 8 d and when plates were fully covered by fungal mycelium, *B*. *xylophilus* were colonized on these plates and allowed to reproduce at 22 °C. To obtain different stages, synchronized L1 and/or L2 eggs were collected and allowed to develop into the L2 juvenile and finally into adults.

The commercial nematicide, abamectin (98.7%), indole, and indole derivatives (listed in Table 1) were purchased from Sigma-Aldrich (St. Louis, USA) and Combi-Blocks (San Diego, CA). All chemicals were dissolved and diluted in 0.1% dimethyl sulfoxide (DMSO). Abamectin in DMSO served as the positive control and 0.1% DMSO was used as the negative control.

### Nematode population inhibition assay

Adult nematodes (~500) were initially treated with 5-iodoindole (0.05 and 0.1 mM) or abamectin (10 µg/mL) and incubated for 24 h at 22 °C as described by Cheng *et al*., 2016^20^. After 24 h, approximately, 10 female and male nematodes were randomly picked and placed at the center of *B*. *cinerea* plates and allowed to grow and reproduce for 8 d. When the *B*. *cinerea* has been completely consumed by nematodes in control plates, nematodes were washed with sterile distilled water by repeated aspiration and recovered from the Petri dishes using the standard micropipette. Nematodes were serially diluted using sterilized distilled water and numbers of nematodes in 100 µL suspensions were counted using an iRiS™ Digital Cell Imaging System (Logos Bio Systems, Korea). The reproduction rates (P_f_/P_i_) (where P_f_ = final nematode population and P_i_ = initial nematode population) of nematodes were calculated. Two trials with six repetitions were performed for this experiment. The data of both trials were combined and presented.

### Locomotor and behavioral assay

*B*. *xylophilus* movement in liquid culture is characterized by stereotypical flexing around the midpoint of the body^20^. Thrashing movements of synchronized juveniles (L2) were monitored by placing them in sterilized distilled water after 24 h treatments with 5-iodoindole (0.05 mM and 0.1 mM) or abamectin (10 µg/mL). The surviving population of nematodes were picked, placed in sterile distilled water and the thrashes were monitored. Numbers of thrashes were counted for 1 minute manually using a handle–held counter; imaging was performed using an iRiS™ Digital Cell Imaging System at a magnification of 10X. A thrash of *B*. *xylophilus* is defined as the movement of head from one direction to another direction and then the same action repeated again. The nematodes that displayed unusual twisting, coiling or remainder motionless for long durations were ignored. Behavioral analyses were conducted at room temperature (22 °C). Two trials with six repetitions were performed for this experiment. The data of both trials were combined and presented.

### Egg deposition assay

Egg deposition assays using one-day-old adult female nematodes were performed in the presence of 5-iodoindole (0.05 mM and 0.1 mM) or abamectin (10 µg/mL) using an established protocol^38^. Briefly, females and males (100 numbers) were treated with 5-iodoindole or abamectin for 24 h in a 96 well polystyrene plate. Approximately each 10 male and female nematodes were picked at random using a sterile platinum wire and transferred to a fresh 30 mm Petri dishes containing distilled water. The number of eggs laid by a single female was recorded after 12 h. Individual female nematodes were analyzed at 12 h and the number of eggs inside the female body was also imaged. The experiments were conducted in three repetitions.

### Embryonic lethality assay

Egg hatching experiments were conducted using established procedures^43^. To estimate hatching rates (HRs), one-day-old adult nematodes (approximately 100 females and 100 males) were transferred to fungal plates and allowed to grow for 24 h. Eggs were then isolated and treated with 5-iodoindole (0.05 mM and 0.1 mM) or abamectin (10 µg/mL) as suspensions in sterilized distilled water. These suspensions (500 µL; approximately 100 eggs) were transferred to the wells of a 24-well tissue culture plate and incubated at 22 °C. L2s were scored after incubation for 24 h but were considered dead if they failed to move when stimulated with a fine platinum wire. Two trials with six repetitions were performed for this experiment. The data of both trials were combined and presented. HR percentages were calculated as follows:

$$\mathrm{HR}( \% )=[{\rm{juveniles}}/({\rm{eggs}}+{\rm{juveniles}})\times {\rm{100}}]{\rm{.}}$$

### Juvenile lethality assay

Juvenile lethality was assessed using a previously established procedure. The nematode eggs were collected and synchronized to obtain L2 stage juveniles by allowing the embryos to hatch in sterile distilled water. Synchronized juveniles (~500 nematodes) were treated with 5-iodoindole (0.05 mM and 0.1 mM) or abamectin (10 µg/mL) and grown on *B*. *cinerea* plates. After 48 h of incubation at 22 °C, nematodes were collected in sterilized distilled water and monitored for the presence of L2, L3 and L4 stages. The presence of L3 and L4 stages indicated juvenile transformation, while the presence of L2 signified an inability to transform. The images were acquired using an iRiS™ Digital Cell Imaging System. Two trials with six repetitions were performed for this experiment. The data of both trials were combined and presented.

The mortality rates (M) of L2s were calculated using Abbott’s formula^59^.

$${\rm{M}}=[{\rm{Mt}}-{\rm{Mc}}/{\rm{100}}-{\rm{Mc}}]\times {\rm{100}}{\rm{.}}$$

where Mt and Mc represent mortality percentages for treated and non-treated controls respectively.

### Molecular docking assay

Molecular docking experiments were conducted as previously described^60^. Docking studies were performed to examine interactions between halogenated indoles or abamectin and the crystal structure of *C*. *elegans* GluCL (glutamate-gated chloride channel) receptor (DOI: 10.2210/pdb4tnv/pdb)^61^, which was retrieved from the RCSB Protein Data Bank (PDB). Ligands (either standard inhibitors (ivermectin and abamectin) or test compounds) were docked into the active sites of GluCL using Schrodinger software 9.3 (Schrodinger Software Solutions, USA). The procedure involved the following critical steps, (i) ligprep, (ii) protein preparation wizard, (iii) glide grid generation and (iv) docking. The grid was generated with close proximity to the active sites and docking was executed using the Glide (grid-based ligand docking energetic) module of Schrodinger 9.3. Interactions were visualized using Incentive PyMOL viewer (v1.8.2.3).

### Seed germination assay

The toxicities of 5-iodoindole and abamectin on seeds were assessed by conducting germination experiments on Murashige and Skoog agar plates^62^. *B*. *oleracea* and *R*. *raphanistrum* seeds were initially soaked in sterile distilled water for a day, washed with 1 mL of 100% ethanol, sterilized with 1 mL of 50% commercial bleach (3% sodium hypochlorite) for 15 min, and rinsed 5 times with 1 mL of sterilized water. Sterilized seeds were then pressed into agar germination plates containing 0.86 g/L (0.2X) Murashige and Skoog medium and 0.7% bacto-agar with or without 5-iodoindole or abamectin. Plates were then incubated at 22 °C and images were captured after incubation for 3 days. Two trials with six repetitions were performed for this experiment.

### Statistical analysis

The trials and repetitions of each experiment are presented in Supplementary Table 1. Values are expressed as means ± SEMs. Statistical significance was determined by pair-wise testing using the Students’ *t*-test. Statistical significance was accepted for p values of <0.05, 0.01, or <0.0001 as indicated.

### Data availability statement

All data generated or analyzed during this study are included in this published article (and its Supplementary Information files).

## Electronic supplementary material

Supplementary Information
