# Supplementary material for: Assessments of iodoindoles and abamectin as inducers of methuosis in pinewood nematode, Bursaphelenchus xylophilus
Source: Sci Rep. 2017 Jul 28;7:6803. doi: 10.1038/s41598-017-07074-2 (PMC5533787; doi:10.1038/s41598-017-07074-2)

Supplementary Information

Assessments of iodoindoles and abamectin as inducers of methuosis in pinewood nematode, *Bursaphelenchus xylophilus*

Satish Kumar Rajasekharan[1], Jin-Hyung Lee[1], Vinothkannan Ravichandran[2], and Jintae Lee[1]*

[1]*School of Chemical Engineering, Yeungnam University, Gyeongsan, 38541, Republic of Korea*

[2]*Shandong University–Helmholtz Institute of Biotechnology, School of Life Science, Shandong University, Jinan, P. R. China.*

Methods

Nematode survival assay.

B. xylophilus fed with *B. cinerea* for 7 days at 25ºC was washed with sterile distilled water, collected and transferred into a 14 ml round tube. Then, nematodes (mixed developmental stages) were further diluted with sterile distilled water to adjust the numbers (about 200 nematodes per 100 μL). After dilution, nematodes were treated with abamectin, indole, or 33 different indole derivatives. Chemical treated nematodes (100 μL) were transferred into 96-well plates and incubated at 22ºC for 24 h. The percentage nematode survival was calculated by counting the number of live and dead nematodes in control and treated groups. Combinatory efficacies of the compounds on *B. xylophilus* survival rates were tested with 5-iodoindole (0.05 mM) and abamectin (10 and 20 μg/mL, separately). The effect of 5-iodoindole on various developmental stages were tested by collecting the nematode eggs and incubating it for specific time period [L2 juveniles (3 days), L3/L4 stages (5 days) and adult nematodes (9 days)]. The trials and repetitions of the experiments are provided in Supplementary Table 1.

Supplementary Table 1. Numbers of experiments performed.

Experiments	Trials	Total repetitions
Screening with indole derivatives (Nematode survival assay)	1	3
Population inhibition assay	2	6
Locomotor and behavioral assay	2	6
Egg deposition assay	1	3
Embryonic lethality assay	2	6
Juvenile lethality assay	2	6
Seed germination assay	2	6

Supplementary Figure S1. Time-lapse images of the locomotor behavior of *B. xylophilus*. Time-lapse images showing the sinusoidal and thrashing movements of L2s in sterile distilled water. (**A**) control, (**B**) abamectin (10 μg/mL), (**C**) 5-iodoindole (0.05 mM), (**D**) abamectin (10 μg/mL), and 5-iodoindole (0.05 mM) in combination. Scale bars = 20 μm.

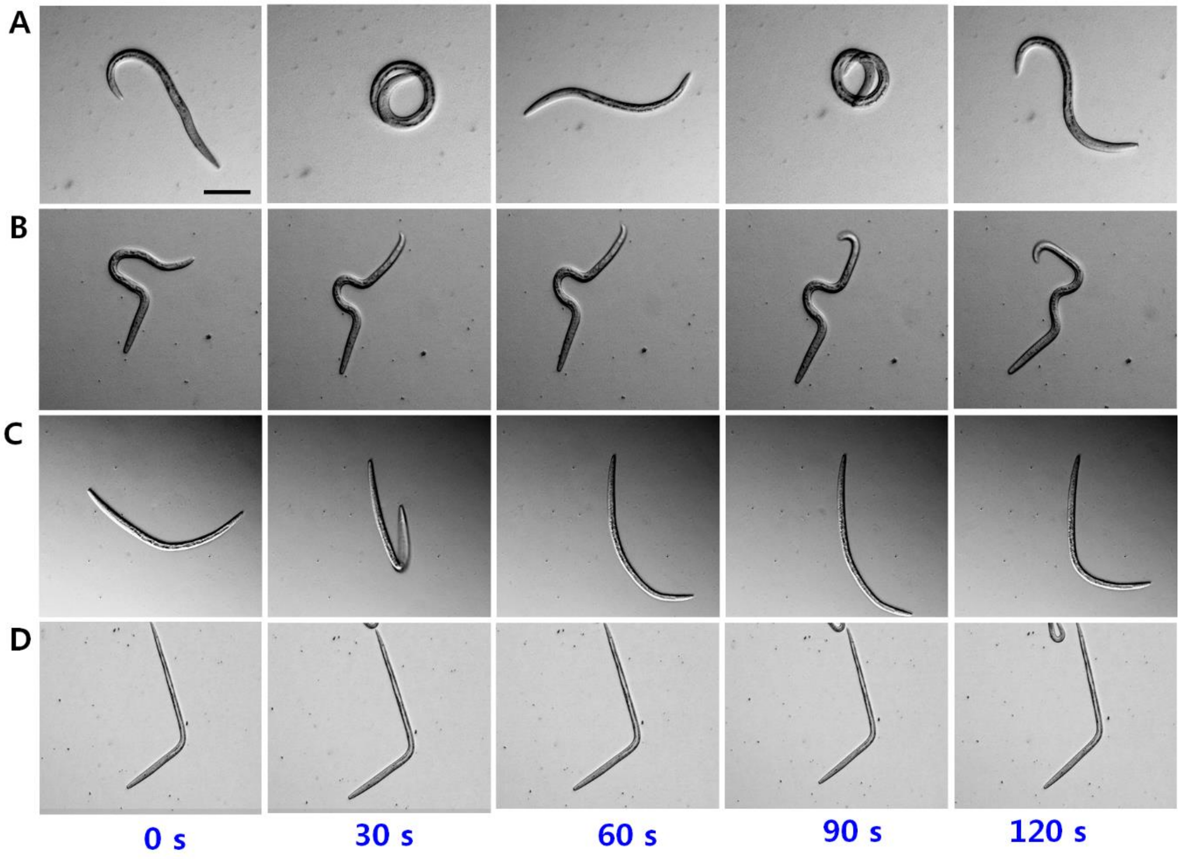

Supplementary Figure S2. Effect of 5-iodoindole on the morphology of L2 eggs and the transparent egg shells. Morphological analysis of L2 stage eggs revealed the presence of multiple vacuoles in 5-iodoindole treated samples. (i) control, (ii) abamectin, (iii and iv) 5-iodoindole, (v) transparent egg shell usually left behind by the L2s during hatching process is shown intact in control groups, and (vi) egg shells in 5-iodoindole treated groups where ruptured and the release of vacuoles and internal contents were distinctly visible. Blue arrows indicate vacuoles and red arrow indicates rupture. Scale bars = 50 µm.

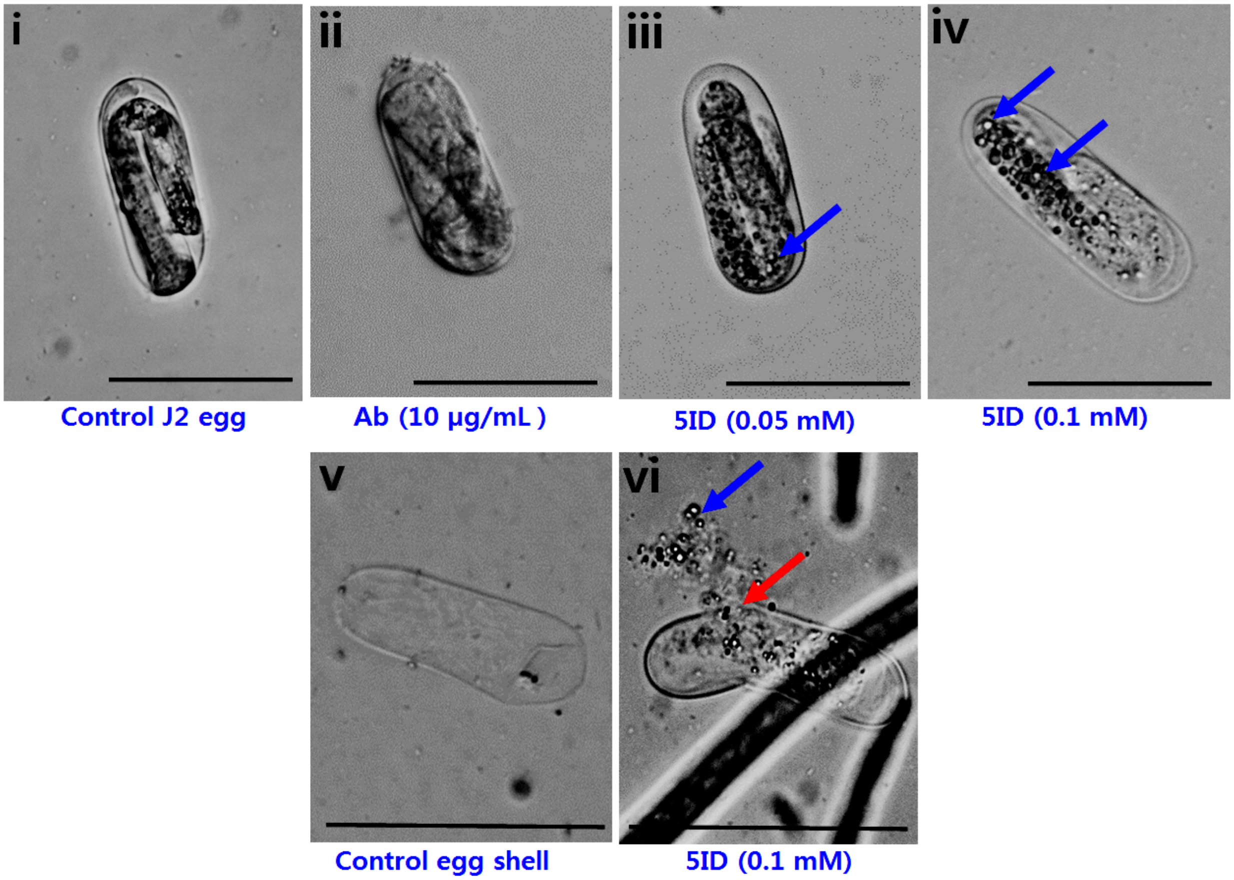

Supplementary Figure S3. Effect of 5-iodoindole on various developmental stages of *B. xylophilus*. Multiple vacuoles (black arrow) were observed in L3, L4, and adult stages. Scale = 50 µm.

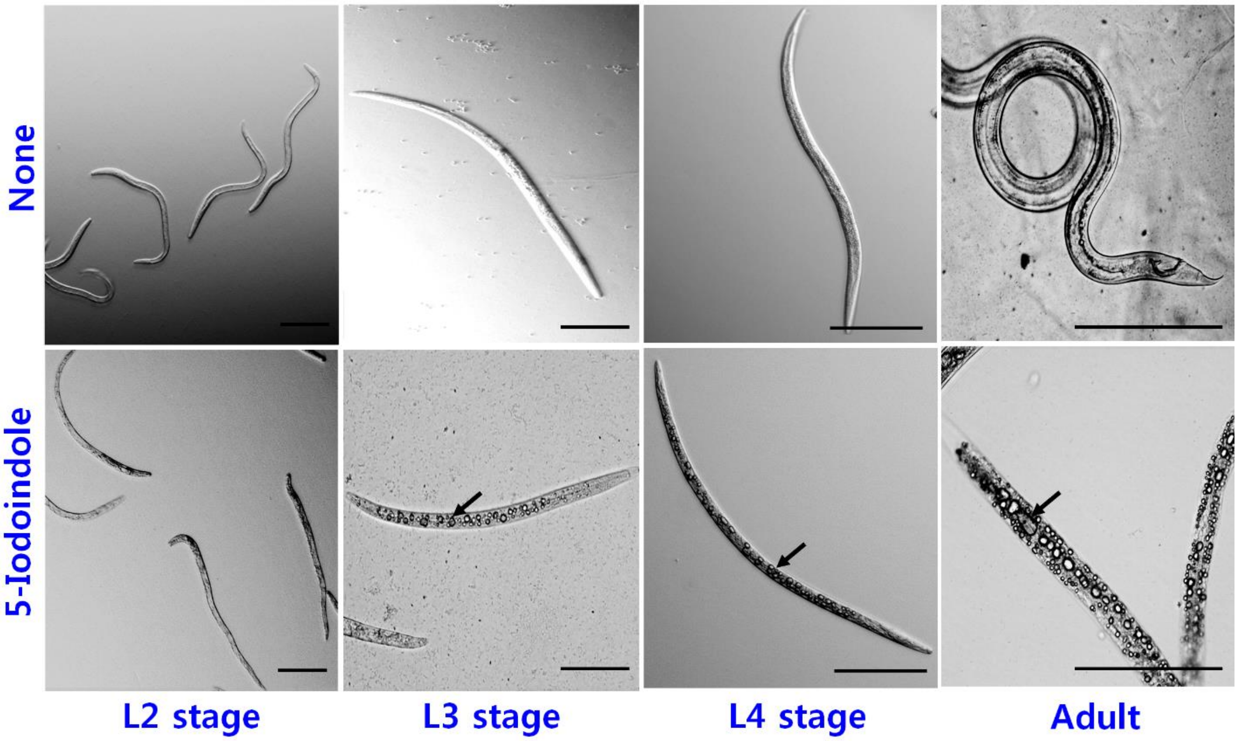

Supplementary Figure S4. Effect of 5-iodoindole on internal structures of *B. xylophilus*. Internal structures of *B. xylophilus* treated with 5-iodoindole (0.1 mM), [(i) control nematode head, (ii) control body part, (iii) control tail portion, (iv) head portion of nematode showing a giant vacuole at its terminus, (v) central body portion of nematode showing multiple vacuoles, and (vi) tail region of nematode showing a giant vacuole and organ shrinkage]. Black arrows in indicate vacuoles, red arrows show internal organelle damage/shrinkage, and blue arrows indicate large voids. Scale bars = 50 μm.

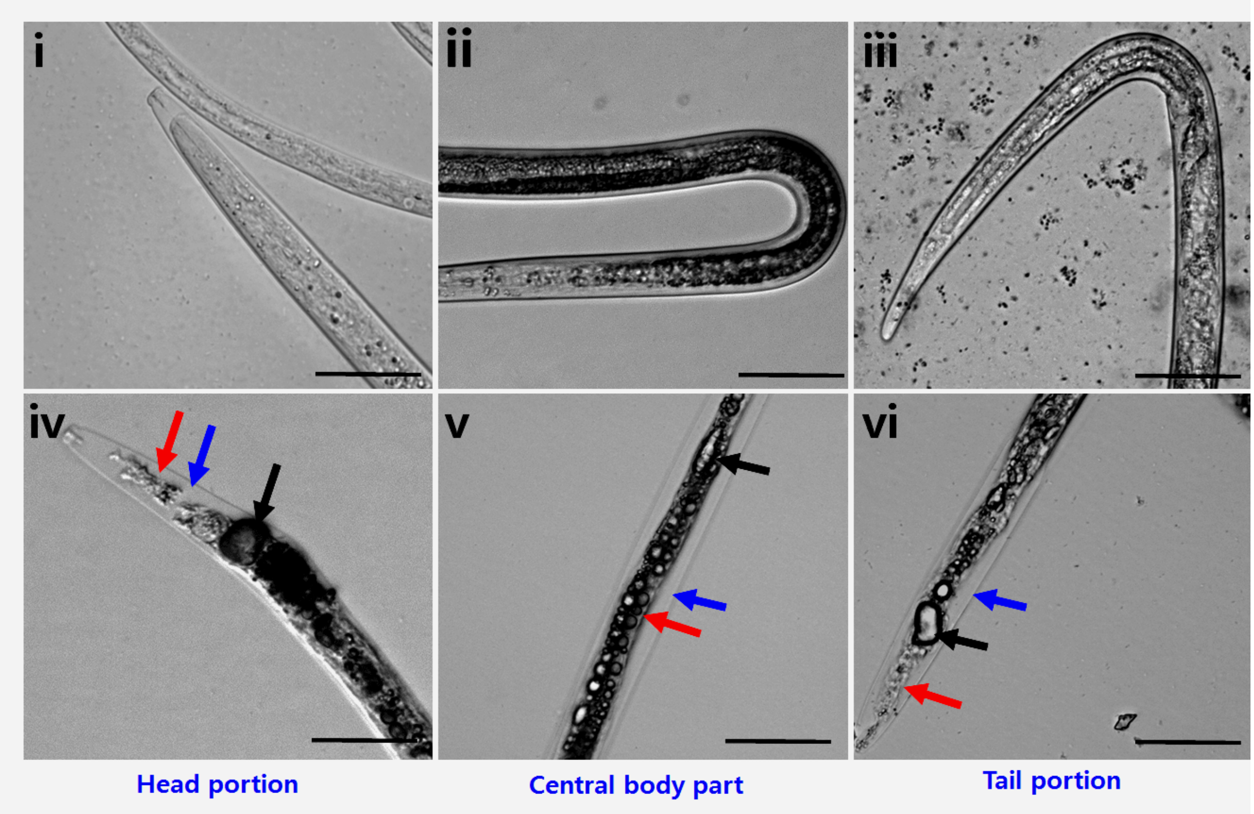

Supplementary Figure S5. Effect of iodide compounds on *B. xylophilus*. Impacts of (**A**) sodium iodide and (**B**) potassium iodide on *B. xylophilus* survival.

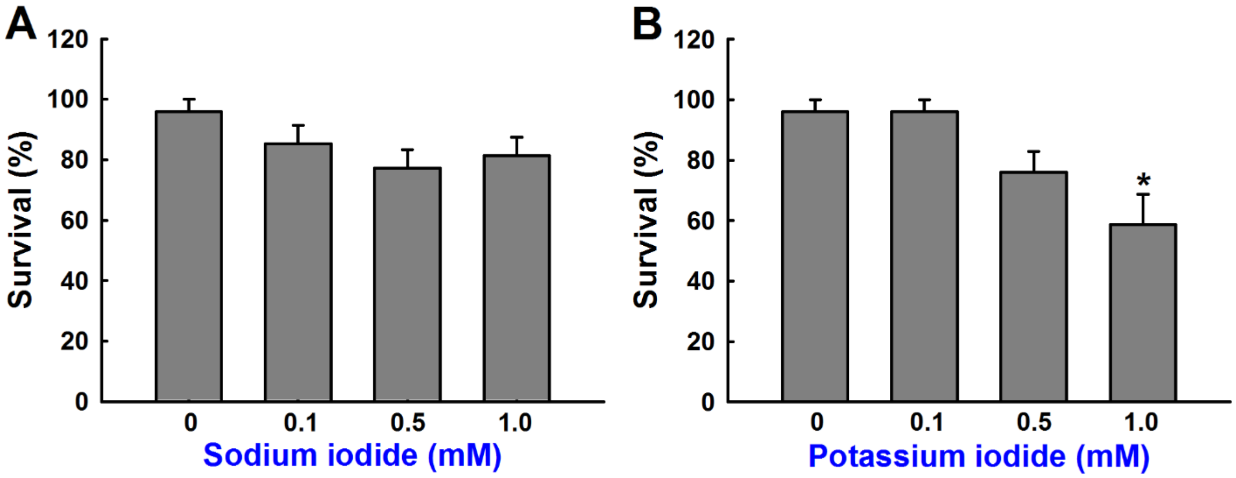

Supplementary Figure S6. Effect of 7-iodoindole on *B. xylophilus*. 7-Iodoindole (2 mM) induced nematode mortality and resulted in the formation of multiple vacuoles in eggs, L3, L4 and adult stage of *B. xylophilus*. Scale bar = 50 µm.

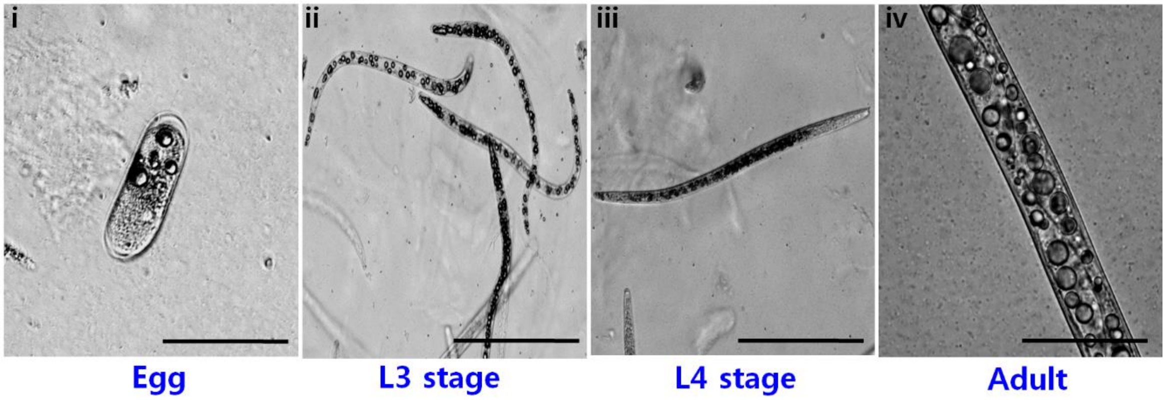

Supplementary Figure S7. Molecular docking of standard nematicides with GluCL receptor. The binding orientations of ligands (**A**) ivermectin and (**B**) abamectin with GluCL active sites. Proteins are shown as ribbons and hydrogen bonds are shown as dotted yellow lines. (**A′**) and (**B′**) show interactions between the respective ligands and the surrounding amino acid residues, pink arrows represent the backbone hydrogen bonds while the dotted pink arrow represent the sidechain hydrogen bonds.

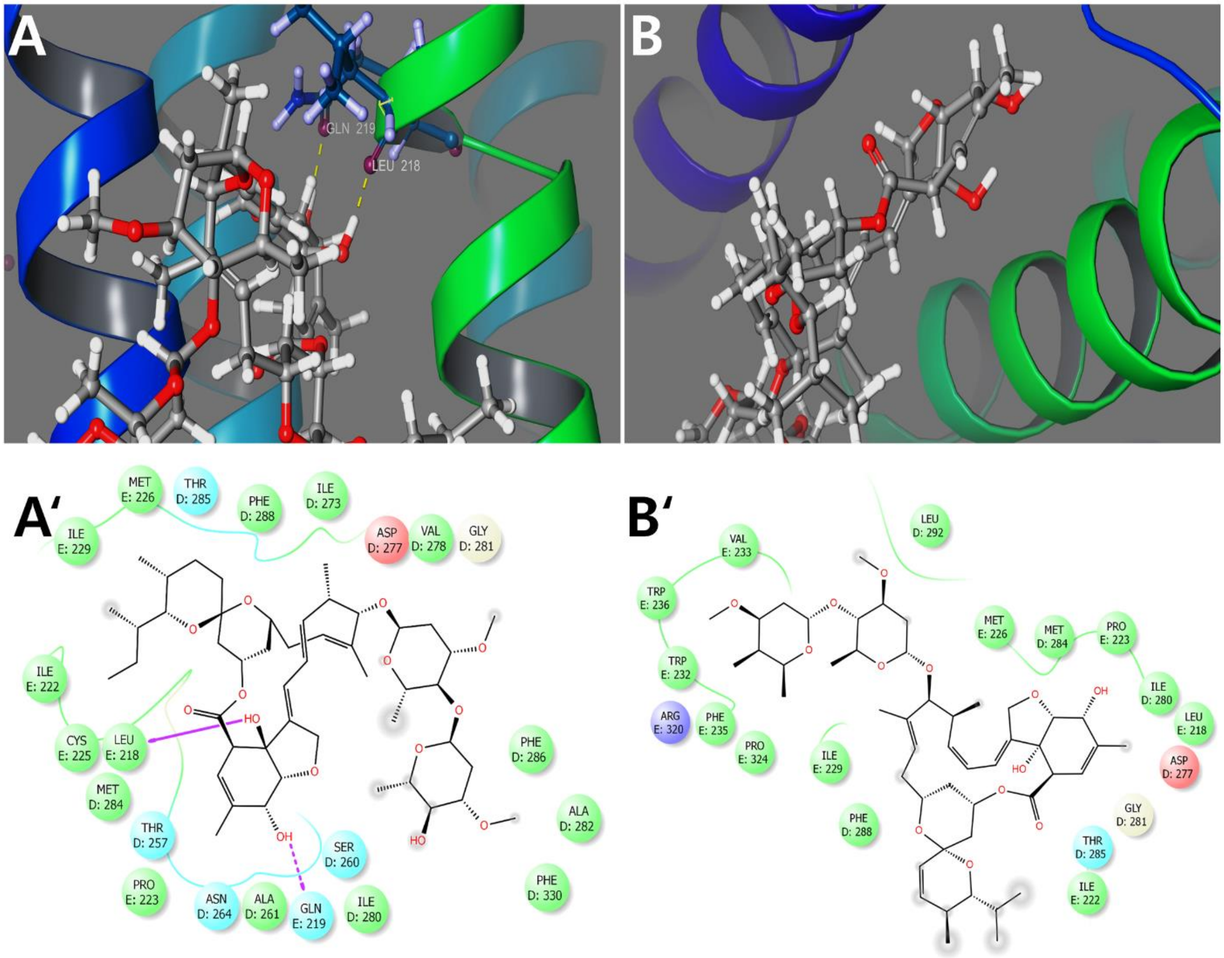

Supplementary Figure S8. Molecular docking of iodoindoles with GluCL receptor. The binding orientations of ligands (**A**) 2-iodondole, (**B**) 3-iodondole, (**C**) 4-iodondole, and (**D**) 6-iodoindole with active sites of the receptor. Proteins are illustrated as ribbons while hydrogen bonds are shown as dotted yellow lines. (**A′**), (**B′**), (**C′**), and (**D′**) show the interactions of respective ligands with the surrounding amino acid residues, pink arrows represent the backbone hydrogen bonds while the dotted pink arrow represent the sidechain hydrogen bonds.

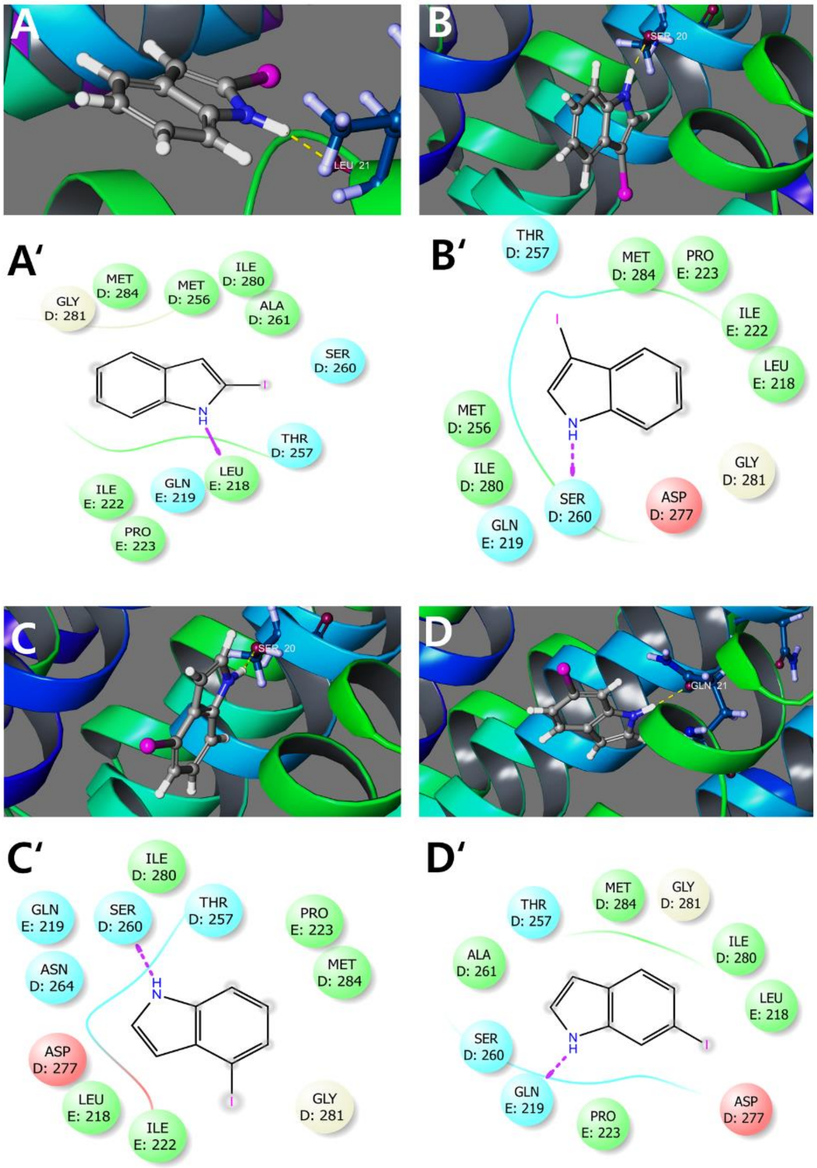

Supplement: Supplementary file 1 — Supplementary Information [file 41598_2017_7074_MOESM1_ESM.pdf]
